# Supplementary material for: Drug repurposing for aging research using model organisms
Source: Aging Cell. 2017 Jun 16;16(5):1006–15. doi: 10.1111/acel.12626 (PMC5595691; doi:10.1111/acel.12626)
Supplement: Supplementary file 7 — Data S1 Zip‐Archive of all report cards. [file ACEL-16-1006-s007.zip › RC_ACE.pdf]

ACE

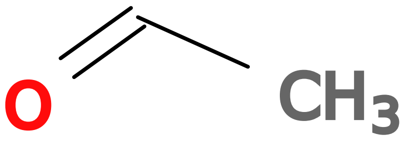

Database identifiers

ChEMBLCompound	CHEMBL170365
CHEBI	15343
eMolecules	474268

Ranking

	Rank	Score
Drosophila	551/697	0.196
C. elegans	413/591	0.129

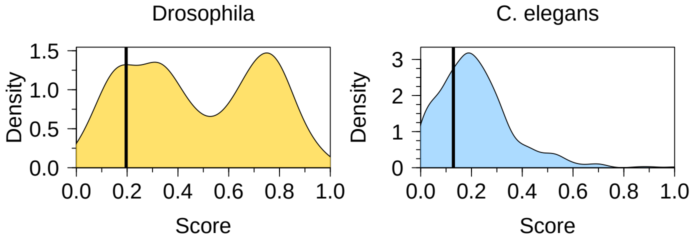

	Ageing implication	Domain conservation	Binding site conservation	Binding affinity	Bioavailability	Lipinski	Promiscuity	Purchasability	Drug approval	**Total**
Drosophila	0.792	0.898	0.747	0.202	(0.9)	0.0	-0.0	0.1	0.0	0.196
C. elegans	0.792	0.888	0.709	0.202	0.29	0.0	-0.0	0.1	0.0	0.129

Names

- **acetaldehyde**
- Ethanal
- ethyl aldehyde

Roles

human metabolite, EC 3.5.1.4 (amidase) inhibitor, carcinogenic agent, mutagen, teratogenic agent, oxidising agent, electron acceptor

Status

Approved drug (according to ChEMBL)	No
Number of Rule of 5 violations	0
Binding affinity to original target in log units (RF-Score prediction)	3.63
Burns *C. elegans* bioavailability prediction	-0.64

Compound Target Characteristics

Neprilysin

Best gene implication in ageing for this target family came from gene P08473 annotated in UniProt release 2014_02. Annotation GO subterm of 7568 (aging) was Inferred from Expression Pattern

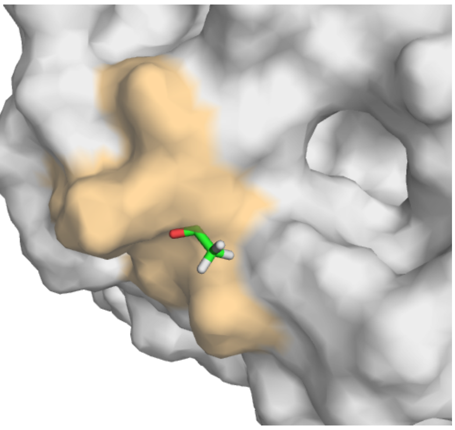

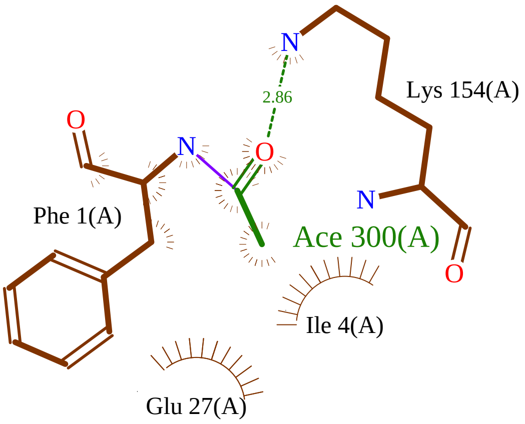

protein	amino acids contacts (binding site)			
PDB:1d19:chainA:P08473	F	I	E	K
sp:P08473:NEP_HUMAN	F	I	E	K
sp:P07861:NEP_RAT	F	I	E	K
sp:Q61391:NEP_MOUSE	F	I	E	K
tr:Q9XZ01:Q9XZ01_DROME	F	F	D	A
tr:Q9W436:Q9W436_DROME	I	V	K	Q
tr:Q9I7I4:Q9I7I4_DROME	F	T	N	E
tr:Q8T062:Q8T062_DROME	F	T	N	E
tr:O44857:O44857_CAEEL	Y	K	R	P
sp:Q18673:NEPL1_CAEEL	S	V	E	A
sp:O16796:NPL11_CAEEL	Y	M	E	P

	whole protein		domain-based		contact-based	
protein	ident	simil	ident	simil	ident	simil
PDB:1d19:chainA:P08473	1.0	1.0	1.0	1.0	1.0	1.0
sp:P08473:NEP_HUMAN	1.0	1.0	1.0	1.0	1.0	1.0
sp:P07861:NEP_RAT	0.94	0.99	0.96	0.99	1.0	1.0
sp:Q61391:NEP_MOUSE	0.94	0.99	0.97	0.99	1.0	1.0
tr:Q9XZ01:Q9XZ01_DROME	0.32	0.71	0.44	0.8	0.25	0.65
tr:Q9W436:Q9W436_DROME	0.34	0.68	0.47	0.83	0.0	0.75
tr:Q9I7I4:Q9I7I4_DROME	0.27	0.58	0.47	0.82	0.25	0.66
tr:Q8T062:Q8T062_DROME	0.26	0.54	0.47	0.82	0.25	0.66
tr:O44857:O44857_CAEEL	0.35	0.74	0.44	0.81	0.0	0.52
sp:Q18673:NEPL1_CAEEL	0.29	0.69	0.45	0.8	0.25	0.39
sp:O16796:NPL11_CAEEL	0.3	0.68	0.43	0.81	0.25	0.71

Nep4 (FBgn0038818) associated phenotypes

locomotor behavior defective

(Information from FlyBase)

nep-2 (UniProt:O44857) annotation

Function: Required for olfactory plasticity, which is the change from positive chemotaxis to dispersal after prolonged exposure to an odorant. Thought to antagonise snet-1 by degrading excess snet- 1 peptides and thus enabling olfactory plasticity. (PubMed:20929849, PubMed:20929849).
Cofactor: Zn(2+) Evidence=(UniProtKB:P08473); Note=Binds 1 zinc ion per subunit. (UniProtKB:P08473);
Subcellular location: Membrane ; Single-pass type II membrane protein
Tissue specificity: Expressed in muscle cells, GLR cells, SMB motor neurons and AIM interneu-

rons. (PubMed:20929849).
Disruption phenotype: Severe defect in olfactory plasticity in response to the odorants benzaldehyde, isoamylalcohol, diacetyl and pyrazine. (PubMed:20929849).

(Information from UniProt)

nep-1 (UniProt:Q18673) annotation
Function: Probable cell surface protease. Required to control the neuronal innervation of pharyngeal pumping. (PubMed:16081104).
Cofactor: Zn(2+)Note=Binds 1 zinc ion per subunit. ;
Subcellular location: Membrane; Single-pass type II membrane protein.
Tissue specificity: Specifically expressed in pharyngeal cells and a single head neuron. (PubMed:16081104).

(Information from UniProt)

nep-2 (UniProt:O16796) annotation
Function: Probable cell surface protease.
Cofactor: Zn(2+)Note=Binds 1 zinc ion per subunit. ;
Subcellular location: Cell membrane ; Single-pass type II membrane protein

(Information from UniProt)
